# Hepatic artery aneurysm post remote pancreaticoduodenectomy

**DOI:** 10.1093/jscr/rjae545

**Published:** 2024-08-28

**Authors:** Fakim Djalal, John Landau, Luc Dubois

**Affiliations:** Division of Vascular Surgery, Schulich School of Medicine and Dentistry, Western University, London, ON, Canada; Division of Vascular Surgery, Schulich School of Medicine and Dentistry, Western University, London, ON, Canada; Division of Vascular Surgery, Schulich School of Medicine and Dentistry, Western University, London, ON, Canada; Department of Epidemiology and Biostatistics, Schulich School of Medicine and Dentistry, Western University, London, ON, Canada

**Keywords:** hepatic artery aneurysm, open repair, pancreaticoduodenectomy

## Abstract

We present a case of a 4.2-cm hepatic artery aneurysm following remote pancreaticoduodenectomy, which extended to the first division of the right hepatic artery. Given the absence of collateral flow from the superior mesenteric artery (SMA) and the inability to place a covered stent, we treated the patient with a saphenous vein graft to the right hepatic artery bifurcation. A CT scan at 1-year demonstrated a patent bypass to the right hepatic artery. We would advise caution when considering hepatic embolization following pancreaticoduodenectomy due to loss of SMA-based collaterals. Techniques that preserve arterial flow should be favored in this situation.

## Introduction

Hepatic artery aneurysms (HAAs) are rare, with an estimated incidence of up to 0.4% [1, 2]. Endovascular techniques (covered stent or coil embolization) are now considered first-line therapy [3]. Here, we report a case of a patient who presented with a large hepatic artery aneurysm years after a Whipple’s procedure. This is a particularly difficult clinical situation as the loss of the gastroduodenal arterial collateral pathway limits embolization options due to concerns of hepatic ischemia; while open repair may be more challenging due to the previous surgery. The patient was treated using open repair and provided written informed consent for the publication of this case.

## Case report

**Figure 1:**
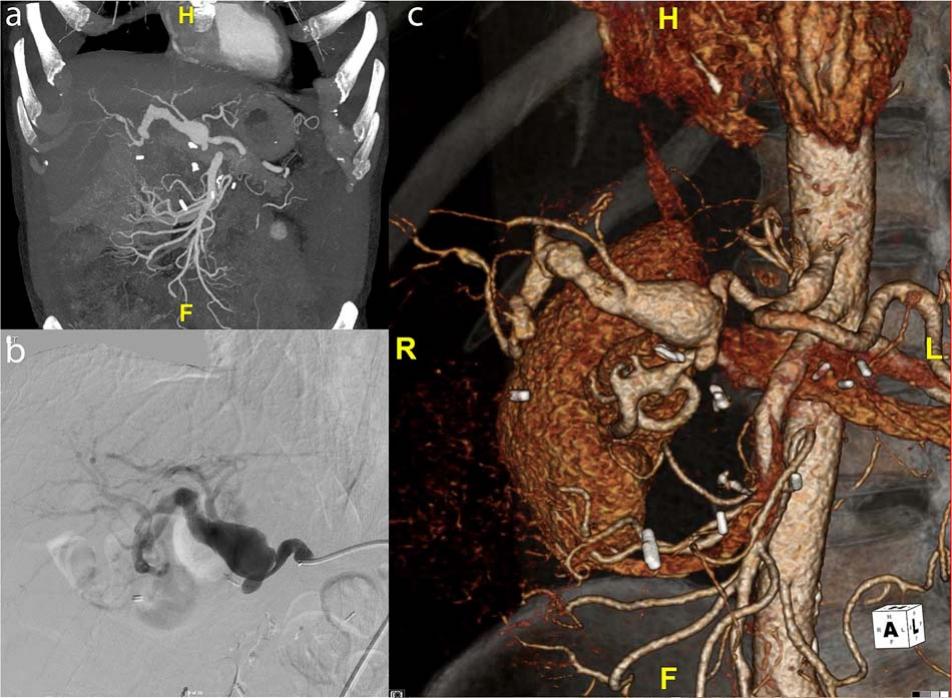
(a) Preoperative CT scan of hepatic artery aneurysm postremote pancreaticoduodenectomy. (b) Catheter-based angiogram of hepatic aneurysm. (c) 3-D reconstruction of hepatic artery aneurysm.

A 59-year-old gentleman was originally referred to vascular surgery in 2011 with a 2.5-cm common hepatic artery aneurysm and was managed conservatively. He had undergone a pancreaticoduodenectomy in 2006 for a presumed pancreatic cancer but was ultimately found to have a benign cyst. The aneurysm was monitored with regular CT scans and grew to 4.2 cm in diameter over the next 9 years and the patient remained asymptomatic. The aneurysm extended from just beyond the origin of the hepatic artery to the first division of the right hepatic artery terminating just before the intraparenchymal portion of the right hepatic artery (Fig. 1a). A catheter-based angiogram was done confirming the extent of the aneurysm and documenting the absence of collateral flow from the superior mesenteric artery (SMA) due to loss of the gastroduodenal artery and other smaller collaterals during the previous Whipple’s procedure (Fig. 1b), which was also evident on 3D reconstruction (Fig. 1c). An endovascular repair was not favored due to the risks of hepatic and biliary ischemia with coiling given the lack of SMA-based collaterals and the inability to place a covered stent. An open repair using a saphenous vein graft was performed.

The previous subcostal extended Kocher incision was reopened; loops of small bowel were mobilized, and the lesser omentum was dissected and opened to expose the celiac trunk. The common hepatic artery was identified and dissected free. The hepaticojejunostomy and bile duct were carefully identified and preserved as the bile duct, and hepaticojejunostomy was located anterior to the mid-body of the aneurysm. In order to facilitate exposure of the distal right and left hepatic arteries, the liver was mobilized from its lateral retroperitoneal attachments to improve mobility of the right lobe. The hepatic artery was controlled form its origin to the first division branches of the right hepatic artery just before it entered the liver parenchyma. A segment of right great saphenous vein was harvested from the thigh. The patient then received 5000 units IV heparin. The aneurysm was opened on either side of the bile duct, taking care to not cause injury to the bile duct and hepaticojejunostomy, which sat anterior to the mid body of the aneurysm and a large amount of thrombus was removed.

**Figure 2:**
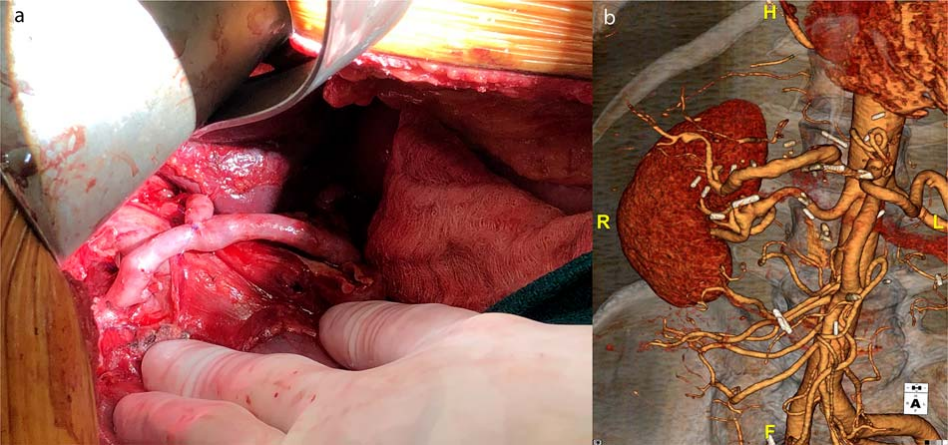
(a) Intraoperative picture of completed saphenous vein bypass to left and right hepatic arteries. (b) One-year postoperative CT scan of patent hepatic artery bypass.

The right hepatic artery was spatulated first as it was the main outflow, and the vein graft was reversed and sewn end-to-end encompassing the orifice of the first branches of the right hepatic artery. The distal end of the vein was spatulated and sewn end-to-end to the common hepatic artery proximal to the aneurysm. The clamps were released, and the right liver was reperfused. We then sewed a separate vein graft off the mid-portion of the vein graft to the left hepatic artery although this artery was quite small 2–3 mm (Fig. 2a).

Post-operatively the patient suffered no changes in biomarkers or any significant complications. He underwent a CT scan, which demonstrated a patent bypass graft prior to discharge on post-operative day 7. A 1-year CT scan demonstrated a widely patent graft to the right hepatic artery but the branch sewn to the left hepatic artery had gone on to thrombose; however, there were no clinical sequelae (Fig. 2b).

## Discussion

Repair of HAA is recommended when the aneurysm reaches 2 cm in asymptomatic patients without significant comorbidities, and 5 cm in patients with significant comorbidities or who are at higher risk for surgery [3]. In this patient with a history of remote pancreaticoduodenectomy, we decided to repair the aneurysm at >4 cm given the higher risk of major morbidity with either an endovascular or open approach. Endovascular techniques are generally preferred if anatomically feasible given the decrease in morbidity and mortality when compared to open repair [3, 4]; however, maintenance of arterial perfusion is recommended and is often provided by SMA-based collaterals when coiling of a hepatic artery aneurysm is undertaken. One recent large single institution series of 11 cases treated with embolization documented no cases of liver ischemia [4]. Given the absence of collaterals in our patient, we were hesitant to offer endovascular coiling for fear of significant liver ischemia. We searched the surgical literature and could not identify a case of a degenerative hepatic aneurysm following a remote Whipple’s procedure to help inform our decision. We did, however, identify reports of significant hepatic ischemia in patients who developed bleeding or pseudoaneurysms of the hepatic artery post-Whipple’s procedure and underwent hepatic artery embolization for hemorrhage control [5–7]. In a series of 18 patients who underwent hepatic artery embolization for delayed hemorrhage following pancreaticoduodenectomy, 67% developed hepatic infarction following embolization, with 1 patient dying of fulminant liver failure [5]. This tangential data supported the need to maintain hepatic artery perfusion in our patient and is what prompted us to recommend open surgical repair. In patients with adequate collateral circulation embolization seems safe but in patients that have undergone pancreaticoduodenectomy, we believe embolization would carry a high risk of clinically significant liver infarction.

## Conclusion

In summary, we present the successful open surgical management of a hepatic artery aneurysm post remote pancreaticoduodenectomy. Although endovascular techniques are favored as first-line therapy for most hepatic artery aneurysms; we would advise caution in embolizing the hepatic artery in patients who have undergone a pancreaticoduodenectomy due to loss of SMA-based collaterals and risk of clinically significant liver ischemia.

## References

[1] Palubinskas S , Rasmussen SL. Hepatic artery aneurysm causing gastrointestinal haemorrhage – case report and literature review. Int J Surg Case Rep 2017;41:12–6. 10.1016/j.ijscr.2017.08.067. 29024839 PMC5742007

[2] Erben Y , De Martino RR, Bjarnason H, et al. Operative management of hepatic artery aneurysms. J Vasc Surg 2015;62:610–5. 10.1016/j.jvs.2015.03.077. 26094044

[3] Chaer RA , Abularrage CJ, Coleman DM, Eslami MH, Kashyap VS, Rockman C, et al. The society for vascular surgery clinical practice guidelines on the management of visceral aneurysms. J Vasc Surg 2020;72:3S–39S, 10.1016/j.jvs.2020.01.039. 32201007

[4] Stark JC , Eisenberg N, Mafeld S, et al. Assessment of open surgical and endovascular management of true hepatic artery aneurysms over 20 years highlights increased rupture risk in females. J Vasc Surg 2022;75:1334–1342.e2. 10.1016/j.jvs.2021.12.054. 34973398

[5] Cho SK , Kim SS, Do YS, et al. Ischemic liver injuries after hepatic artery embolization in patients with delayed postoperative hemorrhage following hepatobiliary pancreatic surgery. Acta Radiol 2011;52:393–400. 10.1258/ar.2011.100414. 21498292

[6] Miura F , Asano T, Amano H, et al. Management of postoperative arterial hemorrhage after pancreato-biliary surgery according to the site of bleeding: re-laparotomy or interventional radiology. J Hepatobiliary Pancreat Surg 2009;16:56–63. 10.1007/s00534-008-0012-3. 19110653

[7] Gwon DI , Ko GY, Sung KB, et al. Endovascular management of extrahepatic artery hemorrhage after pancreatobiliary surgery: clinical features and outcomes of transcatheter arterial embolization and stent-graft placement. AJR Am J Roentgenol 2011;196:W627–34. 10.2214/AJR.10.5148. 21512055

